# Reimers Migration Percentage in Cerebral Palsy Hip Displacement: A Literature-Based Rationale and Statistical Optimization From a Retrospective Cohort

**DOI:** 10.7759/cureus.92795

**Published:** 2025-09-20

**Authors:** Ana L Arenas-Díaz, Geovanny Oleas-Santillán, Fausto Sánchez-Muñoz, Rafael Bojalil, Thania Ordaz-Robles, Silvestre Fuentes-Figueroa, Erika A Barrón-Torres, Clemente Hernández-Gómez, Carlos A Guzmán-Martín

**Affiliations:** 1 Pediatric Orthopaedics, Shriners Hospital for Children Mexico, Mexico City, MEX; 2 Pediatric Orthopaedics, University of San Francisco de Quito (USFQ), Quito, ECU; 3 Department of Physiology, National Institute of Cardiology Ignacio Chávez, Mexico City, MEX; 4 Department of Health Care, Universidad Autónoma Metropolitana-Xochimilco, Mexico City, MEX; 5 Research Programs Department, Shriners Hospital for Children Mexico, Mexico City, MEX; 6 Research Programs Department, Shriners Hospitals for Children Mexico, Mexico City, MEX

**Keywords:** cerebral palsy (cp), hip redislocation, hip surgery, pediatric hip dislocation, reimers’s migration index

## Abstract

Background: Hip redislocation after reconstructive surgery remains a major challenge in children with cerebral palsy (CP), particularly those with severe motor impairment. The Reimers migration percentage (RMP) has long been used empirically to guide surgical decision-making, but the optimal cutoff for predicting redislocation has not been statistically validated.

Objective: This study aimed to determine the most accurate RMP threshold for predicting postoperative redislocation using statistical methods in a retrospective cohort of children with CP.

Methods: We retrospectively analyzed 116 hips from 95 children with CP (Gross Motor Function Classification System (GMFCS) Level IV-V) who underwent reconstructive hip surgery. Preoperative RMP was evaluated using receiver-operating characteristic (ROC) analysis, and the Youden index was applied to identify the optimal cutoff. Hierarchical, block-wise logistic regression analysis that incorporated relevant confounders and surgical variables was performed to assess redislocation.

Results: To statistically determine the optimal RMP cutoff, we first observed that the median preoperative RMP was significantly higher in the Redislocation Group 72.5 (IQR 50-100) compared to the non-redislocation group 50 (IQR 42.25-70, p=0.002). ROC analysis yielded an area under the curve (AUC) of 0.66, with 70% identified as the optimal threshold. In the fully adjusted model, RMP ≥70% remained independently associated with redislocation (adjusted odds ratio (OR) = 3.59, 95% CI 1.45-8.85, p=0.006).

Conclusion: This study provides the first statistical validation of the empirically suggested 70% RMP cutoff for predicting redislocation after reconstructive hip surgery in CP. Although discrimination was modest, the threshold offers a practical tool for risk stratification. Clinically, RMP ≥70% should prompt closer postoperative surveillance and may support consideration of earlier or more extensive reconstructive strategies in high-risk patients.

## Introduction

Hip displacement is a frequent and serious complication in children with cerebral palsy (CP), often resulting in pain, reduced mobility, and the eventual need for complex surgical interventions [1,2]. In 1980, Reimers introduced the Migration Percentage as a radiographic index to quantify hip subluxation in children with neuromuscular disorders, particularly CP. He proposed that hips with Reimers migration percentage (RMP) values exceeding 33% should be classified as subluxated, and those above 90% as dislocated. RMP, calculated as the proportion of the femoral head lying lateral to Perkins’ line, quickly became a cornerstone metric for assessing hip displacement and informing clinical decisions [1].

The prognostic value of RMP was reinforced in the mid-1990s by Miller and Bagg, who demonstrated that children with RMP values above 60% had a high likelihood of progressing to complete dislocation [3]. This evidence catalyzed the integration of RMP thresholds into national hip surveillance programs in countries such as Sweden and Australia. For instance, the Swedish Cerebral Palsy Follow-Up Program (CPUP) adopted the >33% threshold to define “hip displacement,” facilitating timely follow-up and preventive intervention, while the Australian Hip Surveillance Guidelines similarly categorized hips with >30% migration as “at risk” [4-6].

In 2002, Dobson et al. reported that implementing RMP-based surveillance protocols enabled earlier surgical management and substantially reduced the need for salvage procedures in children with CP [7]. This was further supported by Hägglund et al., who observed that using a >33% RMP threshold in systematic screening significantly decreased the incidence of late-stage hip dislocation [8].

Longitudinal studies by Terjesen between 2006 and 2012 confirmed that hip displacement begins early in life and advances rapidly, especially among children with severe motor impairment (GMFCS levels IV and V) [9,10]. Parallel findings by Soo et al. and Wagner & Hägglund revealed that nearly 90% of children with GMFCS level V eventually developed significant hip migration, establishing the importance of GMFCS as a displacement risk stratifier [11,12].

In response to this growing body of evidence, the American Academy for Cerebral Palsy and Developmental Medicine (AACPDM) incorporated RMP thresholds into its official care pathways in 2013, reinforcing the clinical relevance of a 30-33% threshold for intensified monitoring [13]. Building on this, Hermanson et al. developed the CPUP Hip Score in 2015, a predictive tool combining RMP, patient age, and head-shaft angle to estimate the risk of future displacement more precisely [14].

Despite widespread clinical adoption, the empirical validity of commonly used RMP thresholds has been increasingly scrutinized. A 2019 meta-analysis by Agarwal et al. highlighted substantial heterogeneity in cut-off values across studies and emphasized the need for standardized, evidence-based thresholds [15]. These concerns were echoed by Howard et al., who underscored the limited statistical rigor supporting conventional benchmarks [2].

More recently, Minaie et al. (2022) reported that preoperative RMP values exceeding 70% were significantly associated with an increased risk of postoperative redislocation following hip reconstruction, suggesting that higher thresholds may hold stronger predictive value for surgical outcomes [16]. However, their study did not detail the statistical approach used to define this specific cutoff, limiting its interpretability.

Although RMP remains a widely accepted and clinically useful surveillance tool, thresholds such as 33%, 50%, and 90% have primarily been based on historical convention rather than robust statistical validation. Furthermore, their predictive accuracy for postoperative redislocation remains inadequately explored. To address this knowledge gap, the present study applies statistical methodologies to identify the most accurate and clinically meaningful RMP cutoff for predicting postoperative redislocation in children with CP and hip displacement.

## Materials and methods

Study population

This retrospective and analytical study was conducted at Shriners Hospital for Children in Mexico City.

Inclusion criteria

Pediatric patients between 6 and 16 years old, with CP GMFCS IV-V, patients who developed hip subluxation or dislocation and were reconstructed with varus derotational femoral osteotomy (VDRO) ± pelvic osteotomy between Jan 2012 and Dec 2017; minimum 2-year radiographic follow-up; available pre-op anteroposterior (AP) pelvic radiographs meeting AACPDM positioning standards.

Exclusion criteria

Hips with previous reconstructive surgery, inadequate radiographs, <2-year follow-up, or neuromuscular conditions other than CP. We analyzed 116 hips from 95 pediatric patients with spastic CP, all of whom underwent surgical treatment for hip dislocation. Among these, 65 hips experienced redislocation following the initial surgery. To ensure rigorous inclusion criteria, we selected an approximately 1:1 ratio of cases (hips that redislocated) and controls (hips that remained reduced), with near-equal representation of male and female patients, matched by age. Surgical treatment involved either VDRO alone or in combination with pelvic osteotomy. The study protocol received ethical approval from the institutional research ethics committee (Registration Number: CEI-2024-02), in compliance with established ethical standards.

Radiographic assessment

AP pelvis X-ray was performed in all patients with CP. Children were positioned with a horizontal pelvis; the neutral position of the legs and kneecaps was pointing forward. After that, we confirmed a properly positioned AP pelvis X-ray based on symmetric pelvis wings, hip, and femur in a neutral and symmetric oval obturator foramen, and we decided to choose these for their assessment. The radiographical assessment was based on the hip surveillance guidelines by the AACPDM. The RMP was assessed using PACS software by two skilled pediatric orthopedic surgeons in 2022. To prevent bias, observers underwent comprehensive training to standardize measurement methods and interpretation. Intraobserver agreement was tested by having the same observer measure a subset of data repeatedly, ensuring consistency. Our assessments showed high agreement levels, affirming the accuracy and consistency of our measurements. The observer was blinded to the study’s outcome to minimize bias and improve the reliability of the results. Postoperative pelvis radiographs were obtained according to the AACPDM imaging guidelines, with the child in a supine position, lower limbs parallel, and patellae oriented upwards to minimize pelvic obliquity and rotation. Radiographs were considered acceptable when the iliac crests, acetabular margins, and femoral heads were clearly visible. Radiographs with poor quality, including those with excessive pelvic tilt or rotation, incomplete visualization of key landmarks, or motion artifacts, were excluded.

Statistical analysis

A Kolmogorov-Smirnov test was performed to explore the normality of quantitative variables. We used the Mann-Whitney U test to compare quantitative variables between the two groups. An area under the curve (AUC) and a receiver-operating characteristic (ROC) Analysis was performed to determine the discriminative value of RMP, and the optimal cut-off point was estimated through Youden’s index. Additionally, we used the Chi-Square Test and a hierarchical, block-wise logistic regression model for qualitative analysis. The dependent variable was redislocation (case=1) vs no redislocation (control=0). Blocks were entered as follows: Block 1: Age, Sex; Block 2: Topography, GMFCS (IV vs V); Block 3: Open reduction, adductor/psoas release, pelvic osteotomy, femoral VDRO; Block 4: RMP ≥70% (primary exposure). A p-value <0.05 was considered statistically significant and was carried out by SPSS v.26 software.

## Results

**Figure 1:**
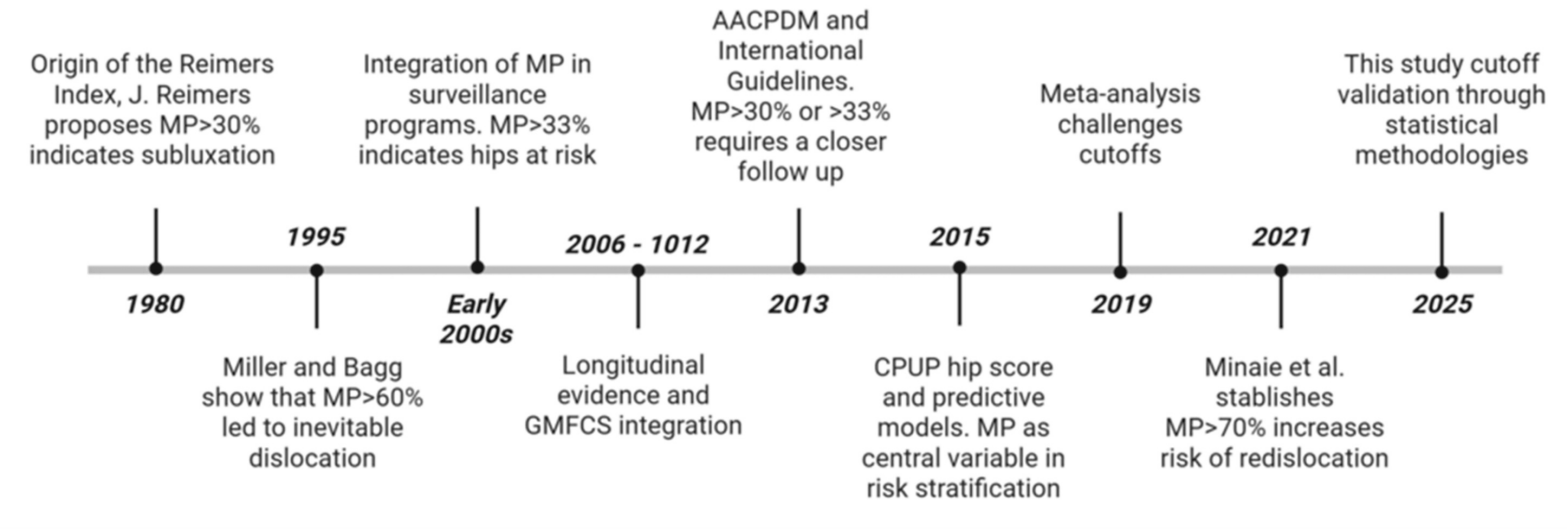
Timeline illustrating the historical development and clinical implementation of the Reimers Migration Percentage in pediatric cerebral palsy. Beginning with its definition by J. Reimers in 1980, the figure highlights major milestones such as its adoption in international surveillance guidelines, validation through longitudinal and predictive studies, and the recent shift toward statistical optimization of cut-off values for surgical decision-making. Notable contributions include the integration of GMFCS levels, the CPUP Hip Score, and recent evidence associating RMP >70% with increased redislocation risk. MP: migration percentage; GMFCS: Gross Motor Function Classification System; AACPDM: American Academy for Cerebral Palsy and Developmental Medicine; CPUP: Cerebral Palsy Follow-Up Program.

A key finding of this study is the comprehensive review of the literature addressing the use of the RMP to evaluate hip displacement in patients with CP. This analysis highlights how the application and interpretation of the RMP have evolved over time, reflecting both advancements in clinical understanding and changes in practice patterns, as illustrated in Figure 1.

The study population had a median age at assessment of 7.5 years, with an interquartile range (IQR) from 6.3 to 8.9 years. Laterality analysis revealed that 61 hips (52.6%) were left-sided. In terms of functional classification, the majority of participants, 90 individuals (77.6%), were categorized as Gross Motor Function Classification System (GMFCS) level IV, while the remaining 26 (22.4%) were classified as GMFCS level V. A comprehensive summary of the clinical and demographic characteristics is provided in Table 1.

**Table 1:** Main characteristics of study population Quantitative Data are represented with median and interquartile range, and qualitative data are shown with frequencies and percentages. Ashworth was measured through the adductor muscles. The following variables were calculated regarding the number of patients (n=96): Age, Weight, Height, Female, Male, Diplegia, Quadriplegia, and GMFCS. The following variables were calculated regarding the number of hips (n=116): Laterality, Ashworth, RMP, Release of adductors and psoas, Open Hip Reduction, VDRO, Pelvic Osteotomy. RMP: Reimers Migration Percentage; GMFCS: Gross Motor Function Classification System; VDRO: varus derotational femoral osteotomy

Clinical Characteristics	All Patients (N= 96) Hips (116)
Age years, median, (IQR)	7.5 (6.3 – 8.9)
Weight kg, median, (IQR)	18.3 (14.5 – 23.8)
Height m, median, (IQR)	1.09 (1.03 – 1.22)
Female, n (%)	50 (52.1)
Male, n (%)	46 (47.9)
Left Hip affected, n (%)	61 (52.6)
Right hip affected, n (%)	55 (47.4)
Diplegia n (%)	40 (41.6)
Quadriplegia n (%)	56 (58.3)
GMFCS IV n (%)	73 (76.04)
GMFCS V n (%)	23 (23.96)
Ashworth ≤ 2 n (%)	50 (43.1)
Ashworth ≥ 3 n (%)	66 (56.9)
RMP, median, (IQR)	61 (50 – 86.5)
Release of adductors and psoas, n (%)	73 (62.9)
Open Hip reduction, n (%)	14 (12.1)
VDRO n (%)	116 (100)
Pelvic osteotomy n (%)	73 (62.9)

**Figure 2:**
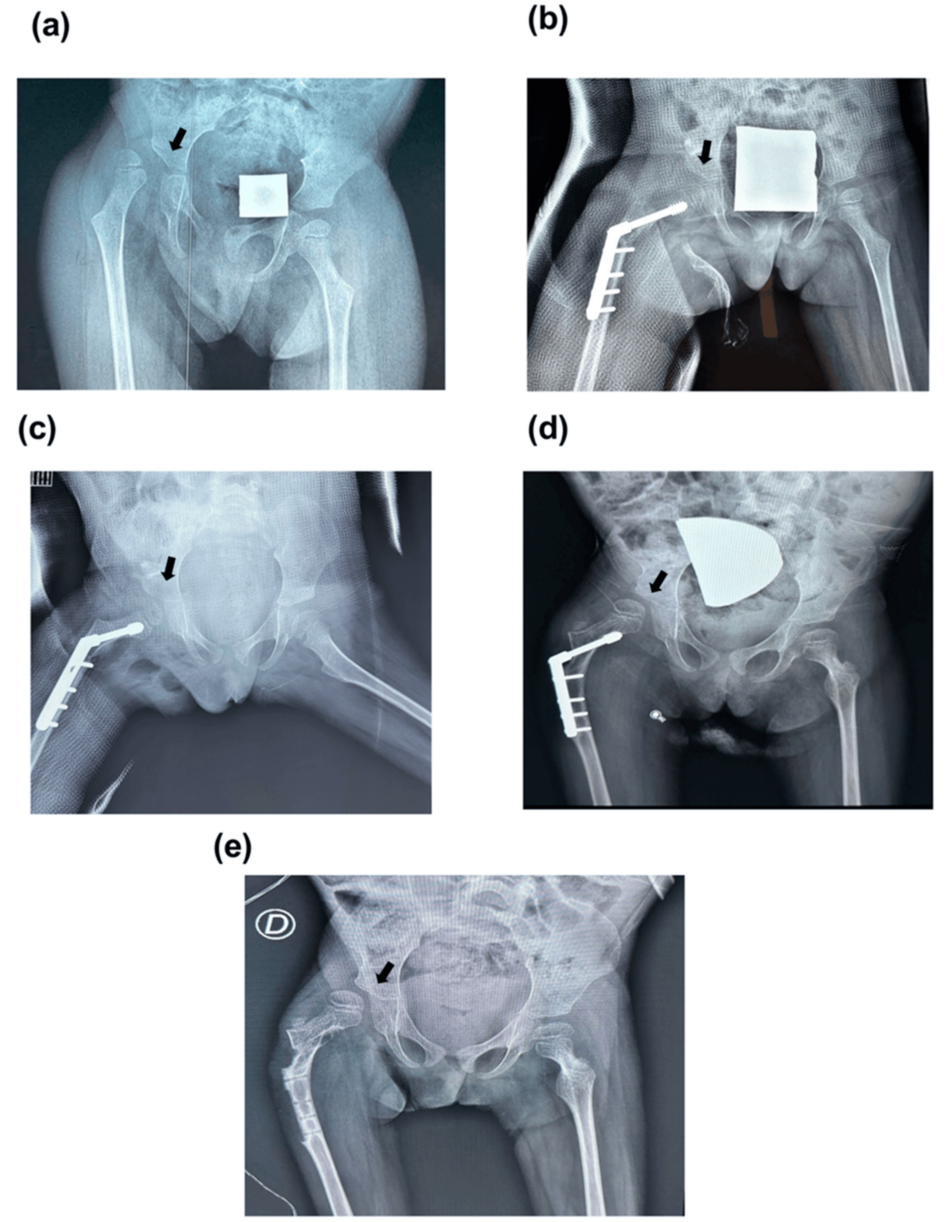
Radiographic representation of a study participant (a) 7- years- old female with spastic quadriplegia and complete dislocation of the right hip. (b) Right hip with VDRO and Pelvic osteotomy. (c) Right hip after six weeks (d). Redislocation of the right hip with an RMP of 50% (e). Right hip with an RMP of 70%. The affected hip is indicated by the black arrow.

Furthermore, we present in Figure 2a sequential radiographic timeline of the right hip in a 7-year-old girl with spastic quadriplegia, capturing the course from initial diagnosis to postoperative complication. First, we show an anteroposterior pelvic view with complete dislocation of the right hip, the femoral head positioned entirely lateral to the acetabulum, consistent with severe hip migration. Following surgical intervention, the immediate postoperative radiograph demonstrates the result of a varus derotation osteotomy combined with a pelvic osteotomy, with fixation hardware in place and the femoral head restored to its anatomic position within the acetabular cavity. At six weeks post-operation, the hip remains reduced, preserving the surgical correction. However, a subsequent image reveals redislocation of the right hip, with an RMP of 50%, indicating partial but clinically significant lateral displacement. In the final stage, the RMP progresses to 70%, reflecting advanced lateral migration and substantial loss of femoral head containment.

**Figure 3:**
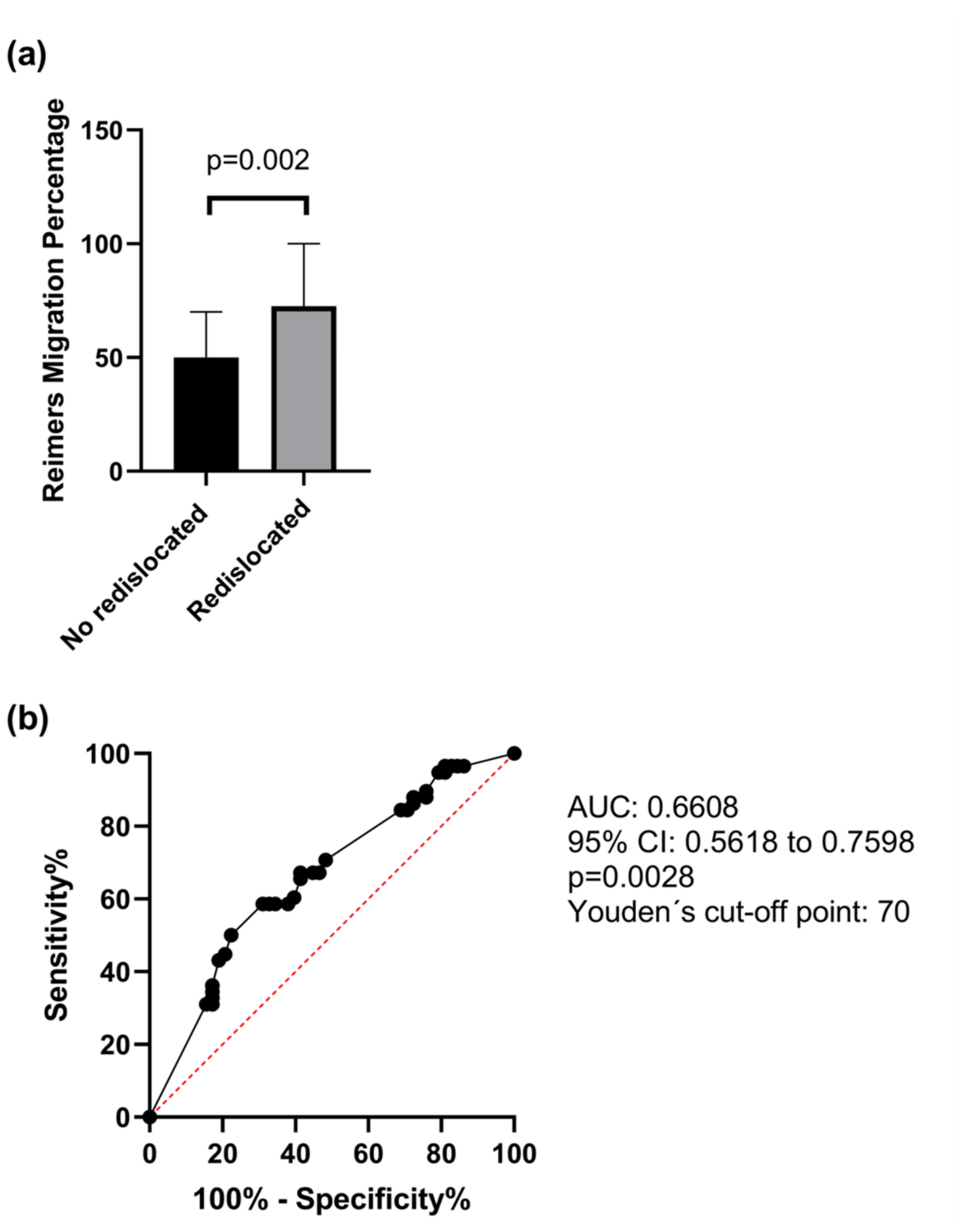
Discriminative value of Reimers Migration Percentage between studied groups. (a) Comparison of quantitative RMP between redislocated and non-redislocated patients through the Mann-Whitney U test. (b) Area under the Curve ROC analysis of RMP and an optimal cut-off point was obtained through Youden’s index.

Then we compared the presurgical Reimers index into two studied groups, the Redislocation Group, comprising patients who experienced hip redislocation following the initial surgery, and the non-redislocation group, consisting of patients who maintained hip stability postoperatively. Comparative analysis revealed that those in the Redislocation Group exhibited significantly higher preoperative RMP values than their non-redislocation counterparts, with a median of 72.5 (IQR: 50 to 100) versus a median of 50 (IQR: 42.25 to 70), a difference that reached statistical significance (p = 0.002) (Figure 3a). To evaluate the discriminatory ability of RMP between these groups, a ROC curve analysis was performed, yielding an AUC of 0.6608 (95% CI: 0.5618 to 0.7598; p = 0.0028). Using the Youden index to identify the optimal threshold, an RMP cut-off value of 70 was determined, balancing sensitivity and specificity for the prediction of postoperative redislocation (Figure 3b).

Subsequently, we stratified participants according to the validated cutoff into two groups: the High RMP Group, comprising patients with preoperative RMP values ≥70%, and the Low RMP Group, with values <70%. To evaluate the association between RMP category and the risk of postoperative hip redislocation, we first applied chi-square tests and then performed logistic regression analyses. In the univariate model, patients in the High RMP Group had a significantly greater likelihood of redislocation, with an OR of 3.15 (95% CI: 1.47-6.75; p=0.002). This association remained significant after adjustment for age and sex, with an OR of 3.03 (95% CI: 1.38-6.64; p=0.008) (Table 2).

**Table 2:** Logistic regression model by using the cut-off point of the Reimers Migration Percentage index for the prediction of redislocation. The table shows the Odds ratios for the dependent variable redislocation, in which we can observe a univariate and multivariate analysis. Multivariate analysis was normalized by age and weight. RMP: Reimers Migration Percentage Index

Dependent Variable: Redislocation after First Surgical Procedure, n=108					
		No redislocation (n= 58)	Redislocation n=58	OR (Univariate)	OR (Multivariate)
Age		8.01± 2.39	7.72 ± 1.76	-	0.907 (0.76 – 1.2) p=0.692
Weight		20.1 ± 9.1	20.63 ± 7.7	-	1.014 (0.76 – 1.2) p=0.622
RMP	<70	40 (68.97)	24 (41.38)	3.15 (1.47 – 6.75) p=0.002	3.026 (1.38 – 6.64) p=0.006
	>70	18 (31.03)	34 (58.62)		

To account for additional patient and surgery-related confounders, we conducted a hierarchical stepwise logistic regression. In the fully adjusted model, RMP ≥70% remained independently associated with redislocation (adjusted OR=3.59, 95% CI: 1.45-8.85; p=0.006). Model performance was acceptable, with good calibration (Hosmer-Lemeshow p=0.483), an overall classification accuracy of 67.2%, and a significant improvement in fit when RMP≥70% was added (Δ−2LL=8.09; χ²(1)=8.09; p=0.004).

Finally, to evaluate the robustness of our findings, we compared the 70% cutoff with alternative thresholds derived from terciles and quartiles (Table 3). Across these stratifications, patients above the respective cutoffs consistently showed a higher risk of hip redislocation, although some comparisons reached only borderline statistical significance. Notably, the cutoff derived from Youden’s index yielded a higher odds ratio (OR) for redislocation, but this result should be interpreted as complementary rather than superior, reinforcing the practical value of the 70% threshold while acknowledging that adjacent cutoffs may also carry predictive relevance.

**Table 3:** Multiple statistical cut-off points compared to predict redislocation. The table presents various statistical cut-off points based on chi-square analysis, assessing risk through odds ratios alongside 95% confidence intervals, aiming to identify the optimal accuracy cut-off point.

Cut-off Point	OR (95% CI)	P-value
Youden´s index (70)	3.14 (1.47 – 6.75)	0.002
Tercile 1 (50)	2.45 (0.99 – 6.04)	0.039
Tercile 2 (75)	3.11 (1.37 – 7.07)	0.005
Quartile 3 (86)	2.34 (0.97 – 5.60)	0.043

## Discussion

CP is the most common cause of physical disability in childhood, characterized by permanent motor dysfunction resulting from a non-progressive lesion in the developing brain [17-19]. Among its many orthopedic complications, progressive hip displacement, through either subluxation or complete dislocation, is one of the most serious and frequent, particularly in non-ambulatory children. This displacement results from spastic muscle imbalance, impaired motor control, and abnormal femoral and pelvic geometry, ultimately leading to pain, loss of function, and a decline in quality of life [20-23].

In this sense, the RMP has long served as the cornerstone metric for assessing hip displacement in children with CP, guiding surveillance strategies and surgical decision-making. Traditionally, thresholds of 33%, 50%, and 90% have been used to classify hips as normal, subluxated, or dislocated, respectively; however, this cutoffs have been based primarily on expert consensus and empirical observations rather than formal statistical validation [1].

Through statistical analysis of a retrospective cohort, we identified a data-driven cutoff that demonstrated superior predictive accuracy compared to conventional benchmarks. This aligns with previous concerns raised in the literature about the empirical limitations of fixed RMP thresholds. For instance, Agarwal KN and collaborators conducted a systematic review revealing substantial variability in how MP values are interpreted across studies, calling for the standardization of this critical parameter [15].

Our study provides the first statistical validation of the 70% RMP threshold for predicting redislocation after reconstructive surgery in children with CP. Through ROC analysis, the Youden index, and multivariable logistic regression, we confirmed that RMP ≥70% independently increased the odds of redislocation. Even after adjusting for age, sex, topography, GMFCS level, open reduction, adductor/psoas release, pelvic osteotomy, and VDRO, the association remained robust (aOR 3.59, 95% CI 1.45-8.85, p=0.006). The model showed good calibration (Hosmer-Lemeshow p=0.483) and a significant improvement in fit when RMP ≥70% was included (Δ−2LL=8.09, p=0.004).

A particularly relevant comparison is the study by Minaie et al., which identified preoperative RMP values above 70% as associated with an increased risk of hip redislocation [16]. While that work first raised awareness of a clinically concerning threshold, the methodology for defining it was not explicitly described. Our study builds upon this by applying formal optimization techniques, specifically ROC curve analysis and hierarchical logistic regression, to statistically validate the 70% cutoff. This approach not only strengthens the evidence base for RMP as a prognostic marker but also enhances its clinical applicability within hip surveillance programs, supporting a shift toward more personalized risk stratification in children with CP.

Clinically, a preoperative RMP ≥70% should be interpreted as a marker of heightened risk that justifies both earlier, more extensive reconstructive strategies (e.g., VDRO with pelvic osteotomy) and closer postoperative surveillance. By statistically validating this empirical threshold, our findings strengthen its role in structured hip surveillance programs and support more individualized risk stratification in children with CP [5,8,10,14].

Finally, to evaluate the robustness of our findings, we compared the 70% cutoff with alternative thresholds derived from terciles and quartiles (Table 3). Across these stratifications, patients above the respective cutoffs consistently showed a higher risk of hip redislocation, although some comparisons reached only borderline statistical significance. Notably, the cutoff derived from Youden’s index yielded a higher OR for redislocation, but this result should be interpreted as complementary rather than superior, reinforcing the practical value of the 70% threshold while acknowledging that adjacent cutoffs may also carry predictive relevance.

This study has several strengths. It leverages a relatively large cohort of GMFCS IV-V patients, applies rigorous statistical validation, and expands prior empirical work (e.g., Minaie et al.) by demonstrating that the 70% threshold withstands adjustment for multiple clinical and surgical factors. The use of hierarchical logistic regression with calibration testing provides additional robustness.

However, several limitations should be considered when interpreting our findings. First, the retrospective design limited the availability of certain clinical variables, such as comorbidities, prior single-event multilevel surgery (SEMLS), and concomitant spine procedures, all of which may have influenced hip stability and contributed to residual confounding. Second, this was a single-center study from Mexico, which may reduce the generalizability of our results to populations with different demographics, healthcare systems, or surgical practices. Third, standardized assessments of muscle tone (e.g., Modified Ashworth Scale) were not consistently available, so we used adductor/psoas release as a pragmatic proxy for spasticity severity. Fourth, surgical heterogeneity was limited, as nearly all hips underwent VDRO, restricting our ability to differentiate the impact of individual procedures. Fifth, precise timing of redislocation events was not available; instead, redislocation status was determined at a minimum follow-up of two years, precluding time-to-event analyses. Finally, the moderate AUC observed in our models indicates that RMP alone is insufficient for comprehensive risk prediction, underscoring the need for multicenter, prospective studies to validate and refine predictive thresholds.

## Conclusions

This study provides the first statistical validation of the 70% RMP cutoff for predicting hip redislocation after reconstructive surgery in children with CP. Using ROC analysis, Youden’s index, and hierarchical logistic regression, we demonstrated that RMP ≥70% independently triples the odds of redislocation, even after adjustment for functional level and surgical variables. Although the predictive strength was moderate, this cutoff offers a practical and clinically relevant tool to guide decision-making.

Clinically, an RMP ≥70% should prompt consideration of earlier and more extensive reconstructive strategies and justify closer postoperative surveillance to allow timely intervention before functional decline occurs. By transforming an empirically suggested threshold into a statistically validated marker, our findings support a more structured and individualized approach to hip surveillance in children with CP. Future multicenter studies are required to confirm the generalizability of this cutoff and to integrate it into comprehensive prediction models that combine radiographic, functional, and surgical risk factors.
